# Characterization of the Roles of SGT1/RAR1, EDS1/NDR1, NPR1, and NRC/ADR1/NRG1 in *Sw-5b*-Mediated Resistance to Tomato Spotted Wilt Virus

**DOI:** 10.3390/v13081447

**Published:** 2021-07-25

**Authors:** Zhengqiang Chen, Qian Wu, Cong Tong, Hongyu Chen, Dan Miao, Xin Qian, Xiaohui Zhao, Lei Jiang, Xiaorong Tao

**Affiliations:** Key Laboratory of Plant Immunity, Department of Plant Pathology, Nanjing Agricultural University, Nanjing 210095, China; 2017202019@njau.edu.cn (Z.C.); 2018202018@njau.edu.cn (Q.W.); 2018202016@njau.edu.cn (C.T.); 2015202014@njau.edu.cn (H.C.); 2019102033@njau.edu.cn (D.M.); qian_xin6@163.com (X.Q.); zhaoxiaohui_g@163.com (X.Z.); jianglei062x@ahau.edu.cn (L.J.)

**Keywords:** tomato spotted wilt virus, *Sw-5b*, NLR receptor, plant innate immunity, defense signaling

## Abstract

The tomato *Sw-5b* gene confers resistance to tomato spotted wilt virus (TSWV) and encodes a nucleotide-binding leucine-rich repeat (NLR) protein with an N-terminal Solanaceae-specific domain (SD). Although our understanding of how *Sw-5b* recognizes the viral NSm elicitor has increased significantly, the process by which *Sw-5b* activates downstream defense signaling remains to be elucidated. In this study, we used a tobacco rattle virus (TRV)-based virus-induced gene silencing (VIGS) system to investigate the roles of the SGT1/RAR1, EDS1/NDR1, NPR1, and NRC/ADR1/NRG1 genes in the *Sw-5b*-mediated signaling pathway. We found that chaperone SGT1 was required for *Sw-5b* function, but co-chaperone RAR1 was not. *Sw-5b*-mediated immune signaling was independent of both EDS1 and NDR1. Silencing *NPR1*, which is a central component in SA signaling, did not result in TSWV systemic infection in *Sw-5b*-transgenic *N. benthamiana* plants. Helper NLR NRCs (NLRs required for cell death) were required for *Sw-5b*-mediated systemic resistance to TSWV infection. Suppression of NRC2/3/4 compromised the *Sw-5b* resistance. However, the helper NLRs ADR1 and NRG1 may not participate in the *Sw-5b* signaling pathway. Silencing ADR1, NRG1, or both genes did not affect *Sw-5b*-mediated resistance to TSWV. Our findings provide new insight into the requirement for conserved key components in *Sw-5b*-mediated signaling pathways.

## 1. Introduction

Plants have evolved different layers of defense against pathogen infection [1]. In the first layer, plants use an extracellular pattern recognition receptor (PRR) to recognize a pathogen-associated molecular pattern (PAMP) to trigger PAMP-triggered immunity (PTI). In response, plant pathogens use multiple virulence effectors to suppress PTI. As a second layer of defense, plants have evolved various intracellular nucleotide-binding leucine-rich repeat (NLR) receptors to recognize these effectors and invoke effector-triggered immunity (ETI). NLR proteins are the largest group of resistance genes in plants [2]. Plant NLRs typically have a variable N-terminal domain: a Toll/interleukin-1 receptor (TIR) domain or a coiled-coil (CC) domain, a central NB-ARC (nucleotide binding adaptor and APAF-1, R proteins, and CED-4) domain, and a C-terminal leucine-rich repeat (LRR) domain [3,4]. Hereafter, NLRs with CC domains are referred to as CNLs, and NLRs with TIR domains are referred to as TNLs. Effector-mediated NLR activation results in a robust defense response that is typically associated with a hypersensitive response (HR) and constrains pathogens at the site of infection [1,4,5,6]. However, our understanding of how NLRs activate downstream resistance remains limited. To date, only a few common key downstream immune-signaling components of NLR-mediated resistance have been identified.

Tomato spotted wilt virus (TSWV, genus *Orthotospovirus*; family *Tospoviridae*) infects more than 1000 different plants in at least 85 families [7] and is among the most destructive plant viruses [8,9,10], causing more than 1 billion USD in crop losses worldwide each year [11]. The *Sw-5b* gene is the most effective resistance gene for controlling TSWV in tomato [12,13,14,15]. Since it was introgressed from Peruvian tomato, *Solanum peruvianum* L., it has been used widely in tomato-breeding projects. *Sw-5b* encodes a protein belonging to a CNL-type resistance gene. It specifically recognizes the movement protein NSm of TSWV [16,17,18]. *Sw-5b* has also been found to confer broad-spectrum resistance against various American-type tospoviruses by recognizing a highly conserved 21 amino-acid region in NSm (NSm21) [19]. *Sw-5b* carries an extended N-terminal Solanaceae-specific domain (SD) and develops a multilayered regulatory mechanism to control its autoinhibition and activation [20]. For activation, *Sw-5b* uses a two-step recognition mechanism involving both SD and the LRR domain in the detection of viral NSm [21]. This two-step recognition increases the specificity of NSm recognition and also significantly enhances the sensitivity of NSm detection. Although our understanding of how *Sw-5b* recognizes viral elicitor NSm has increased significantly, the process by which *Sw-5b* activates downstream defense signaling has yet to be elucidated.

SGT1 (suppressor of the G2 allele of skp1) has been found to be required for the induction of the immunity mediated by many NLRs [22,23]. As a protein conserved in eukaryotes, SGT1 functions in a variety of biological processes by interacting with different protein complexes [24,25,26]. It regulates the protein accumulation levels of NLR immune receptors [27] and participates in the nucleocytoplasmic distribution of tobacco NLR protein N [28]. SGT1 is associated with RAR1, which is required for Mla12 resistance, and heat-shock protein 90 (HSP90), forming a molecular chaperone complex that is required for appropriate accumulation of NLR proteins [23]. Although SGT1 is required for the establishment of the *Sw-5b*-mediated HR [29], the requirement for SGT1/RAR1/HSP90 in *Sw-5b*-mediated induction of systemic resistance to TSWV has not been tested.

EDS1 (enhanced disease susceptibility 1) is a lipase-like protein that is needed for all tested TNLs to activate downstream signaling. EDS1 is also necessary for some CNLs, such as the CNL HRT and the CC R-protein RPW8 [30,31]. However, some CNLs, such as RPS2, RPM1, and RPS5, require an alternate protein, NDR1 (non-race-specific disease resistance 1), for immunity [32]. The requirement for EDS1/NDR1 in the *Sw-5b*/TSWV resistance pathways has not yet been tested.

Salicylic acid (SA) plays an important role in many NLR-mediated resistance mechanisms. In *Arabidopsis*, NPR1 (non-expresser of pathogenesis-related genes 1) is a central component in SA signaling [33,34]. NPR1 was found to be a receptor of SA [35]. It controls the transcription of approximately 90% of the SA-dependent genes in *Arabidopsis* [33,36,37,38]. NPR1 shuttles into the nucleus, where it interacts with TGA transcription factors, thereby activating defense genes to establish plant immunity [39]. Tobacco NPR1 is required for N-mediated resistance to tobacco mosaic virus (TMV) [37]. However, *HRT*-mediated resistance to turnip crinkle virus (TCV) in *Arabidopsis* is independent of NPR1 [40]. The role of NPR1 in the *Sw-5b* resistance pathway is not known.

Recently, many “sensor” NLR proteins have been found to require “helper” NLR proteins to activate downstream immune signals [41,42]. Three types of “helper” NLR proteins have been described in plants: the NRC (NB-LRR protein required for HR-associated cell death) family, ADR1 (activated disease resistance 1) family, and NRG1 (N requirement gene 1) family. All of these are CNL-type proteins [42,43]. The NRC family proteins in tobacco and tomato are required for the induction of HR mediated by a variety of NLRs [29,41,44]. Although NRC2/3/4 have been shown to be required for the induction of HR mediated by *Sw-5b* [29], whether they are required for induction of systemic resistance to TSWV remains to be elucidated. ADR1 family proteins that function downstream of RPS4/RRS1, RPP2, SNC1, CHS1/SOC3, RPP4, and RPS2 are involved in both TNL- and CNL-mediated immunity [45,46,47,48]. NRG1 was first identified in *Nicotiana benthamiana* L. using virus-induced gene silencing (VIGS) and was found to be required for *N*-mediated resistance to TMV [49]. NRG1 is essential for *Roq1*, *N*, and *RPP1*-mediated HR and disease resistance [50]. *Ry_sto_*-dependent extreme resistance to potato virus Y also requires NRG1 [51]. *NRG1* is likely a conserved key component of TNL-mediated immune signaling pathways. The *ADR1* and *NRG1* gene families are function redundant, and their N-terminal domains are related to RPW8-CC [46]. Disease susceptibility was enhanced in an *adr1 adr1-L1 adr1-L2 nrg1.1 nrg1.2 nrg1.3* sextuple helperless mutant in comparison to *adr1* triple and *nrg1* triple mutants [47]. *ADR1* and NRG1 were also found to be involved in *Rx2*-mediated resistance to potato virus X (PVX) [46]. However, the requirement for ADR1 and NRG1 in *Sw-5b*/TSWV has not yet been tested.

In this study, we characterized the requirement for *SGT1*/*RAR1*, *EDS1*/*NDR1*, *NPR1*, and *NRC*/*ADR1*/*NRG1* genes in the *Sw-5b*-mediated immune signaling pathway using a TRV-based virus induced gene silencing (VIGS) approach. We found that SGT1, but not RAR1, was required for *Sw-5b*-mediated resistance. *Sw-5b* function was independent of both EDS1 and NDR1. Suppression of *NPR1* did not cause TSWV systemic infection in *Sw-5b*-transgenic *N. benthamiana* plants. The helper NLRs NRC2/3/4, but not ADR1 and NRG1, were required for *Sw-5b*-mediated systemic resistance to TSWV infection. These findings provide new insights into the requirement for conserved components in the *Sw-5b*-mediated signaling pathway.

## 2. Materials and Methods

### 2.1. Plasmid Construction

The cDNA fragments corresponding to *Sw-5b* (AY007366.1), *NbHSP90* (Niben101Scf15166g03015.1), *NbRAR1* (LC314308.1), *NbEDS1* (Niben101Scf06720g01024.1), *NbNDR1* (AY438029.1), *NbNPR1* (Niben101Scf14780g01001.1), *NbNRC2a* (KT936525.1), *NbNRC2b* (KT936526.1), *NbNRC3* (MK692736.1), *NbNRC4* (MK692737.1), *NbNRG1* (DQ054580.1), and *NbADR1* (Niben101Scf02422g02015.1) were amplified by polymerase chain reaction (PCR) using PrimeSTAR High-Fidelity DNA Polymerase (TaKaRa, Dalian, China) and cloned into pTRV2 [37]. For constructing TRV-*NbNRC2/3/4* and TRV-*NbNRG1/NbADR1*, gene fragments were fused by overlap PCR. The pTRV2-*NbPDS*, pTRV2-*NbSGT1*, and pTRV2-*GUS* vectors have been described previously [52].

### 2.2. Host Plant and Virus

Four to 6 week old *N. benthamiana* plants were used for the VIGS experiments. The source of the TSWV Yunnan isolate has been described previously [53]. TSWV Yunnan isolates were propagated in *N. benthamiana*. For long-term preservation, fresh systemically infected leaves of *N. benthamiana* were stored in a refrigerator at −80 °C. Plant leaves containing TSWV were ground in 0.01 M phosphate buffer (pH 7.4). The *Sw-5b*-transgenic *N. benthamiana* line has been described previously [20]. The VIGS-treated and TSWV-inoculated plants were maintained in a growth chamber at a temperature of 24 °C under a 16 h light/8 h dark photoperiod.

### 2.3. VIGS Assay

pTRV1, pTRV2, and their derivatives were individually introduced into *Agrobacterium* strain GV3101 cells by electroporation. Agrobacterium cultures were resuspended in agroinfiltration buffer (10 mM MgCl_2_, 10 mM MES pH 5.6, and 100 μM acetosyringone) and adjusted to a final concentration of OD_600_ = 0.5. The *Agrobacteria* containing TRV RNA1 and RNA2 were mixed at a 1:1 ratio and incubated for 3 to 5 h in the dark at 28 °C. The mixtures were co-infiltrated into fully expanded leaves of *N. benthamiana* using a 1 mL needleless syringe. Six plants were used for each treatment and/experiment. Each experiment was repeated three times.

### 2.4. Total RNA Isolation and Quantitative RT-PCR Analysis

Total RNAs were extracted from *N. benthamiana* plant leaves using an RNA Easy Fast Isolation kit (catalog no. DP452, Tiangen, Beijing, China). First-strand cDNAs were synthesized using Oligo-dT primer and M-MLV Reverse Transcriptase (Promega, Madison, WI, USA). The expression of genes silenced by TRV-based VIGS was quantified on an ABI 7500 Real-Time PCR system (Life Technologies, Carlsbad, CA, USA) using Power SYBR Green Master Mix (Life Technologies). All of the primers used for quantitative reverse transcriptase (qRT)-PCR are listed in Supplementary Table S1. *NbActin* and *NbEF1a* were used as internal controls in the assayed samples.

### 2.5. Western Blot Assays

Total proteins were extracted from *N. benthamiana* plant leaves in extraction buffer (10% glycerol, 25 mM Tris-HCl, pH 7.5, 150 mM NaCl, 1 mM EDTA, 10 mM DTT, 2% polyvinylpolypyrrolidone, 1× protease inhibitor cocktail, 0.2% TritonX-100). The crude plant extracts were centrifuged at 12,600× *g* for 10 min, and the supernatant was mixed with 3 × SDS loading buffer (150 mM Tris-HCl, pH 6.8, 6% SDS, 0.3% bromophenol blue, 30% glycerol, 300 mM DTT) and boiled for 10 min. Protein samples were separated in 10% SDS-PAGE gels and then transferred to PVDF membranes. The blots were probed with an anti-TSWV N antibody followed by HRP-conjugated goat anti-rabbit antibodies (1:10,000). An ECL Substrate Kit (Thermo Scientific, Hudson, NH, USA) was used to develop the blots. A Bio-Rad ChemiDoc Touch imaging system (Bio-Rad, Hercules, CA, USA) was used to visualize the signals.

## 3. Results

### 3.1. Silencing Sw-5b by TRV-Based VIGS Abolished the Resistance of Sw-5b-Transgenic N. benthamiana to TSWV Infection

**Figure 1:**
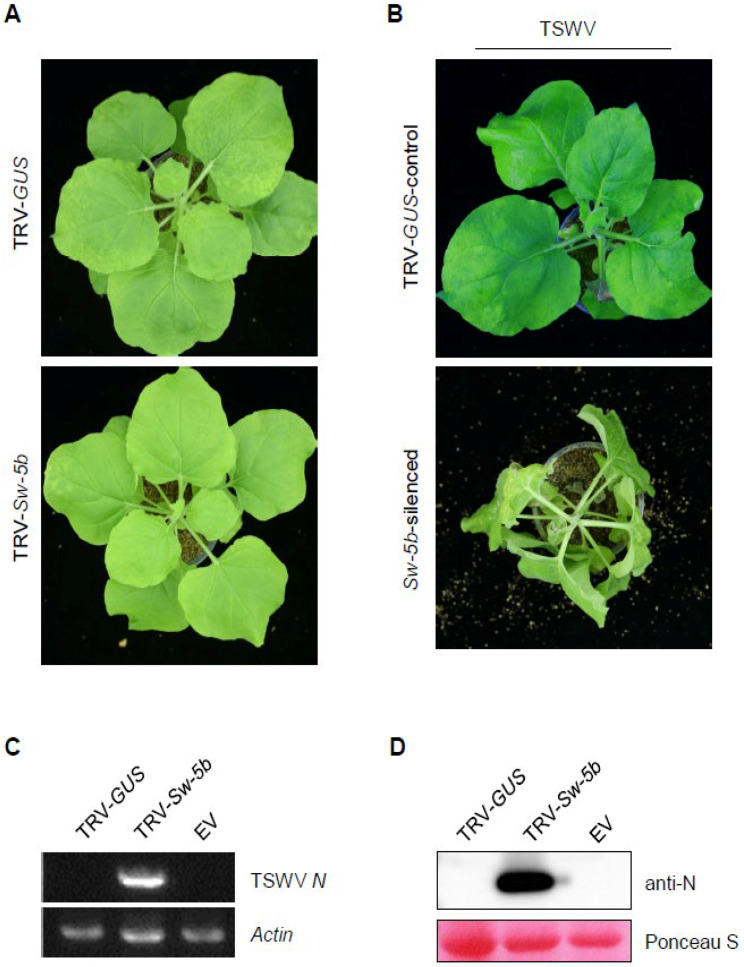
Silencing *Sw-5b* through VIGS abolished the resistance of *Sw-5b*-transgenic *N. benthamiana* to TSWV infection. (**A**) *Sw-5b*-transgenic *N. benthamiana* plants treated with TRV-*GUS* and TRV-*Sw-5b*. *Agrobacterium* harboring TRV1 was mixed equally with *Agrobacterium* harboring TRV2 with gene fragments of *GUS*, or *Sw-5b*, and the mixture was co-infiltrated into 4–6 week old leaves of *Sw-5b*-transgenic *N. benthamiana*. The treated plants were photographed at 3 weeks post TRV infection. (**B**) Analysis of TSWV systemic infection of *Sw-5b*-transgenic *N. benthamiana* plants pretreated with TRV-*Sw-5b*. TRV-*GUS* treatment was used as the control. The newly emerged leaves of *Sw-5b*-transgenic *N. benthamiana* plants silenced for *Sw-5b* or pretreated with TRV-*GUS* at 3 weeks post TRV infection were inoculated with crude extract of TSWV-infected tissues. The plants were photographed at 14 days post TSWV inoculation. The systemic infection was evident in all 18 *Sw-5b*-silenced plants in three repeated experiments. (**C**) RT–PCR analysis of the expression of TSWV N RNA in systemic leaves of *Sw-5b*-transgenic *N. benthamiana* plants pretreated with TRV-*GUS* or TRV-*Sw-5b*. The internal reference gene was *NbActin*. (**D**) Western blot analysis of TSWV N accumulation in systemic leaves of *Sw-5b*-transgenic *N. benthamiana* silenced for *Sw-5b* and pretreated with TRV-*GUS* control at 10 dpi post TSWV inoculation. A plant sample expressing empty vector (Vec.) was used as a negative control. Ponceau S staining of Rubisco was used as a protein loading control.

Our laboratory previously generated *Sw-5b*-transgenic *N. benthamiana* that confer resistance to TSWV infection [20]. To investigate the downstream defense signaling of *Sw-5b*-mediated resistance, we adopted a TRV-based VIGS to silence downstream candidate genes in *Sw-5b*-transgenic *N. benthamiana* plants, followed by TSWV infection. Before silencing the downstream candidate genes, we first tested silencing the *Sw-5b* gene in *Sw-5b*-transgenic plants using a TRV-based VIGS approach, and we examined the process of TSWV infection in the *Sw-5b* silenced plants. A 500 bp fragment of the *Sw-5b* gene was amplified by PCR and inserted into the TRV2 vector. As a control, a fragment from the *GUS* gene was inserted into the TRV2 vector. TRV2 carrying a fragment from *NbPDS* was also used as a control. *Agrobacterium* carrying TRV1 mixed equally with *Agrobacterium* carrying TRV2-*Sw-5b*, TRV2-*GUS*, or TRV-*NbPDS* was co-infiltrated into the leaves of 4–6 week old *Sw-5b*-transgenic *N. benthamiana* plants. At 3 weeks post TRV treatment, newly emerged leaves of *Sw-5b* transgenic *N. benthamiana* infected with TRV-*NbPDS* were photobleached (Figure S1A), implying that *NbPDS* was silenced. At this stage, the corresponding newly emerged leaves of *Sw-5b*-transgenic *N. benthamiana* plants pre-treated with TRV-*Sw-5b* or TRV-*GUS* control (Figure 1A and Figure S1B) were inoculated with crude sap from TSWV-infected tissue. At 14 days post TSWV inoculation (dpi), typical viral symptoms including leaf curling, stunting, and systemic wilt were observed in the *Sw-5b*-transgenic *N. benthamiana* silenced for *Sw-5b* (Figure 1B). No such symptoms were observed in the plants treated with the TRV-*GUS* control (Figure 1B).

Total RNA was extracted from systemic leaves of TSWV-inoculated transgenic *N. benthamiana* treated with TRV-*Sw-5b* or TRV-*GUS.* The RT-PCR analysis showed that the TSWV *N* gene was detected in systemically infected leaves of the *Sw-5b*-silenced transgenic *N. benthamiana*, but not in the TRV-*GUS*-treated control plants (Figure 1C). Western blotting assays further confirmed that TSWV N protein accumulated in the systemically infected leaves of *Sw-5b*-transgenic plants treated with TRV-*Sw-5b*, but not in those treated with the TRV-*GUS* control (Figure 1D). These results imply that silencing *Sw-5b* by TRV-based VIGS abolished *Sw-5b*-mediated resistance to TSWV infection in transgenic *N. benthamiana* plants.

### 3.2. SGT1, But Not Rar1, Was Required for Sw-5b-Mediated Resistance to TSWV

SGT1, RAR1, and HSP90 are required for the induction of disease resistance mediated by a variety of NLR proteins. They form a molecular chaperone complex that is important for regulating the protein stability of plant NLRs [54,55].

**Figure 2:**
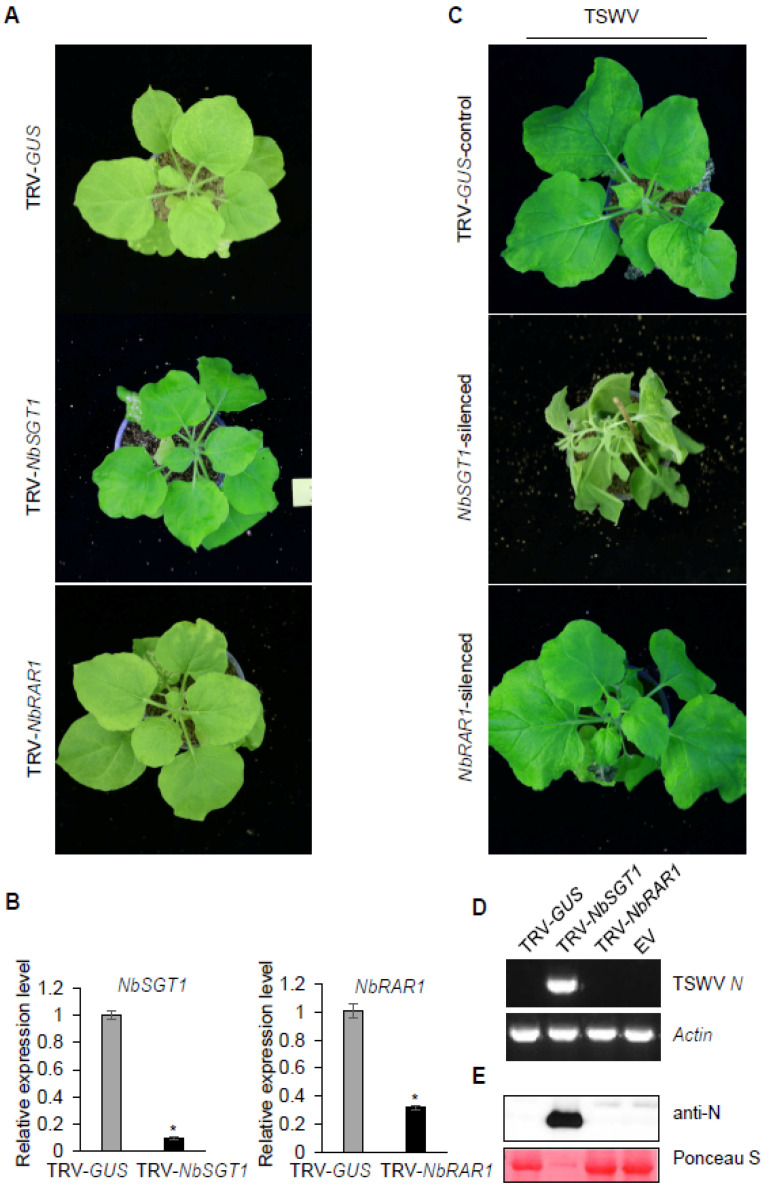
Silencing expression of *SGT1*, but not *RAR1*, compromised the *Sw-5b*-mediated resistance to TSWV. (**A**) *Sw-5b*-transgenic *N. benthamiana* plants infected with TRV-*NbSGT1* and TRV-*NbRAR1* at 3 weeks post TRV treatment. TRV-*GUS* treatment was used as a control. *Agrobacterium* harboring TRV1 was mixed equally with *Agrobacterium* harboring TRV2-*NbSGT1* or TRV2-*NbRAR1* and co-infiltrated into leaves of 4–6 week old *Sw-5b*-transgenic *N. benthamiana*. The TRV-treated plants were photographed at 3 weeks post agroinfiltration. (**B**) Quantitative RT-PCR analysis of the expression of *NbSGT1* and *NbRAR1* mRNA in the newly emerged leaves of TRV-treated transgenic *N. benthamiana* plants at 3 weeks post agroinfiltration. The *NbActin* and *NbEF1a* genes were used as internal reference genes. Asterisks indicate significant differences (Student’s *t*-test, * *p* < 0.05). (**C**) Analysis of TSWV systemic infection of *Sw-5b*-transgenic *N. benthamiana* plants silenced for *NbSGT1* and *NbRAR1.* TRV-*GUS* was used as a control. The gene-silenced newly emerged leaves of *Sw-5b*-transgenic *N. benthamiana* plants were inoculated with crude extract of TSWV-infected tissues. The TSWV challenged plants were photographed at 14 days post viral inoculation. The systemic infection was evident in all 18 *NbSGT1*-silenced plants. (**D**) RT–PCR detection of TSWV *N* RNA in systemic leaves of *Sw-5b*-transgenic *N. benthamiana* plants silenced for *NbSGT1* and *NbRAR1* at 14 days post TSWV inoculation. The internal reference gene was *NbActin*. (**E**) Western blot analysis of TSWV N protein accumulation in systemic leaves of *Sw-5b*-transgenic *N. benthamiana* plants silenced for *NbSGT1* and *NbRAR1* using a TSWV N-specific antibody. The leaves were collected at 14 days post TSWV inoculation. A plant sample expressed with p2300 empty vector (Vec.) was used as a negative control. Ponceau S staining was used as a protein loading control.

To examine whether SGT1, RAR1, and HSP90 play essential roles in *Sw-5b*-mediated resistance to TSWV, we used TRV-based VIGS to silence *NbSGT1*, *NbRAR1*, and *NbHSP90* in *Sw-5b*-transgenic *N. benthamiana*. TRV-*GUS* was used as a negative control. At 3 weeks post TRV treatment, in comparison with the TRV-*GUS*-treated controls, the transgenic *N. benthamiana* plants treated with TRV-*NbSGT1* and TRV-*NbRAR1* did not exhibit significant growth defects (Figure 2A and Figure S2). However, the transgenic *N. benthamiana* treated with TRV-*NbHSP90* were deformed, with extremely small newly emerged leaves and inhibited growth, making it difficult to inoculate them further with TSWV (Figure S3A). The qRT-PCR results showed that mRNA expression of *NbSGT1*, *NbRAR1*, and *NbHSP90* was significantly reduced in newly emerged leaves of these transgenic plants (Figure 2B and Figure S3B). The newly emerged leaves of transgenic *N. benthamiana* silenced for *NbSGT1* or *NbRAR1* were inoculated with TSWV crude extracts from systemically infected tissues. At 14 days post TSWV inoculation, the TSWV spread to the systemic leaves, causing symptoms including systemic wilting in *NbSGT1*-silenced plants (Figure 2C). No systemic infection was observed in the TRV-*GUS*-treated control plants (Figure 2C). In the *Sw-5b* transgenic *N. benthamiana* silenced for *NbRAR1*, to our surprise, neither TSWV systemic infection nor viral symptoms were observed (Figure 2C). RT-PCR assays showed that the TSWV *N* gene was detected in the systemically infected leaves of *NbSGT1*-silenced transgenic *N. benthamiana*, but not in the TRV-*NbRAR1* and TRV-*GUS* pretreated plants (Figure 2D). Western blotting assays further confirmed that the TSWV N protein accumulated in the systemically infected leaves of *NbSGT1*-silenced transgenic *N. benthamiana* plants, but not in the leaves of TRV-*NbRAR1-* or TRV-*GUS*-treated plants (Figure 2E). These findings imply that SGT1, but not RAR1, is involved in *Sw-5b*-mediated resistance to TSWV.

### 3.3. Suppression of EDS1 and NDR1 Did Not Disrupt Sw-5b-Triggered Immunity

**Figure 3:**
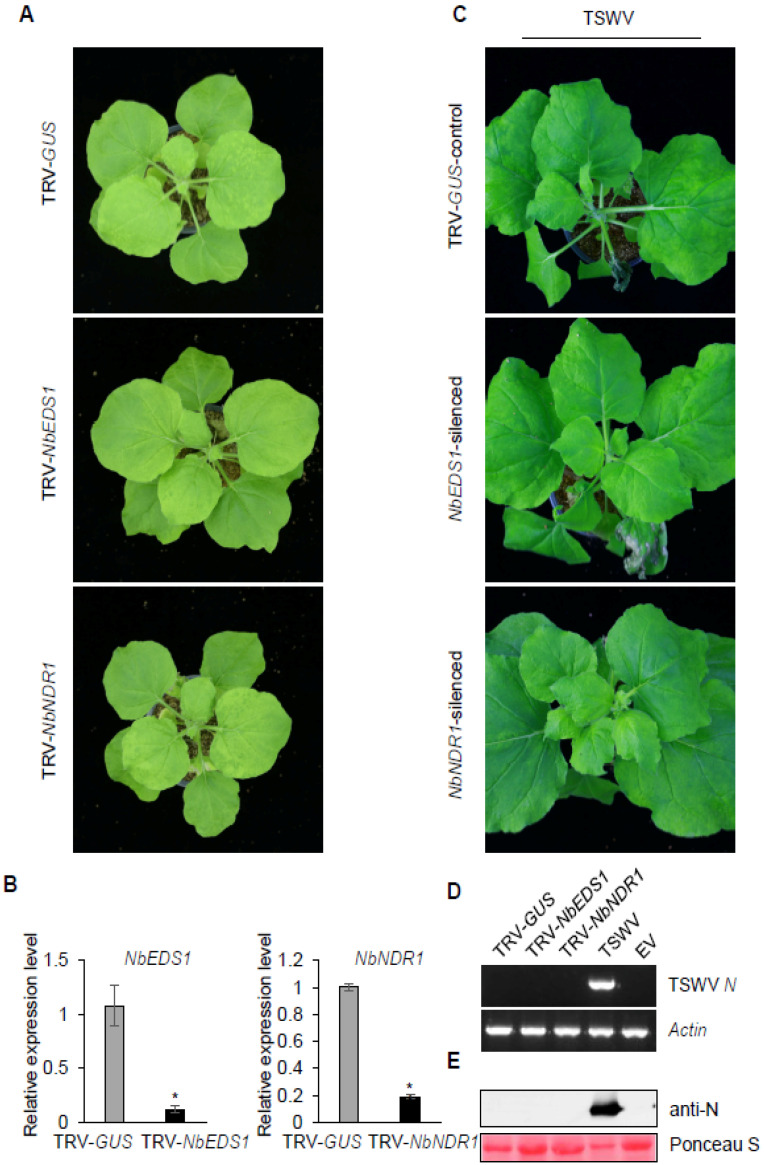
*Sw-5b*-mediated resistance to TSWV was independent of *EDS1* and *NDR1.* (**A**) *Sw-5b*-transgenic *N. benthamiana* plants treated with TRV-*NbEDS1* and TRV-*NbNDR1*. TRV-*GUS* was used as a control. *Agrobacterium* harboring TRV1 was mixed equally with *Agrobacterium* harboring TRV-*NbEDS1* or TRV-*NbNDR1* and co-infiltrated into leaves of 4–6 week old *Sw-5b*-transgenic *N. benthamiana*. The TRV-treated plants were photographed at 3 weeks post agroinfiltration. (**B**) qRT-PCR analysis of expression of *NbEDS1* and *NbNDR1* mRNA in newly emerged leaves of *Sw-5b*-transgenic *N. benthamiana* treated with TRV-*NbEDS1*, TRV-*NbNDR1*, or TRV-*GUS*. Samples were collected at 3 weeks post TRV treatment. Values were normalized using *NbActin* and *NbEF1a* genes as a reference. Student’s *t*-test was used for statistical analysis (* *p* < 0.05). (**C**) Analysis of TSWV systemic infection in *Sw-5b*-transgenic *N. benthamiana* plants silenced for *NbEDS1* and *NbNDR1.* TRV-*GUS* was used as the control. The gene-silenced newly emerged leaves of *Sw-5b*-transgenic *N. benthamiana* plants were inoculated with crude extract of TSWV-infected tissues. The plants challenged with TSWV were photographed at 14 days post inoculation. (**D**) RT–PCR analysis of expression of TSWV *N* RNA in systemic leaves of *Sw-5b*-transgenic *N. benthamiana* plants silenced for *NbSGT1* and *NbRAR1* at 14 days post TSWV inoculation. A TSWV-infected *N. benthamiana* sample was used as a positive control. The reference gene was *NbActin*. (**E**) Western blot analysis of TSWV N protein accumulation in systemic leaves of *Sw-5b*-transgenic *N. benthamiana* plants silenced for *NbSGT1* and *NbRAR1* in panel C using specific antibodies against N. A plant sample expressed with p2300 empty vector (Vec.) was used as a negative control. Ponceau S staining was used as a protein loading control.

EDS1 is a common signal component in the TNL-mediated ETI process [56], in which it plays an important role downstream of TIR NADase activity [57,58]. EDS1 is also involved in the immunity mediated by some CNLs [30,31]. NDR1 is a key signal component of the plant immune response mediated by some CNLs [59,60,61]. To investigate whether EDS1 and NDR1 are involved in *Sw-5b*-mediated resistance to TSWV infection, *Agrobacterium* cultures containing TRV1 were mixed with *Agrobacterium* carrying TRV-*NbEDS1* or TRV-*NbNDR1* and co-infiltrated into leaves of *Sw-5b*-transgenic *N. benthamiana.* The TRV-*GUS* was used as a control. At 3 weeks post TRV treatment, the transgenic *N. benthamiana* treated with TRV-*NbEDS1* and TRV-*NbNDR1* did not show any differences compared to the plant treated with the TRV-*GUS* control (Figure 3A and Figure S4). qRT-PCR confirmed that expression of *NbEDS1* and *NbNDR1* mRNA transcripts in the corresponding silenced plants was significantly reduced (Figure 3B). Subsequently, gene-silenced newly emerged leaves of transgenic *N. benthamiana* plants were inoculated with TSWV. At 14 days post TSWV inoculation, no obvious virus systemic infection was observed in the *NbEDS1*- or *NbNDR1*-silenced transgenic *N. benthamiana* plants (Figure 3C). RT-PCR and Western blot assays confirmed that TSWV was absent in the systemic leaves of the *NbEDS1*- and *NbNDR1*-silenced transgenic *N. benthamiana* plants (Figure 3D,E). These results imply that silencing expression of the *NbEDS1* or *NbNDR1* genes did not disrupt the resistance of *Sw-5b*-transgenic *N. benthamiana* plants to TSWV infection.

### 3.4. Silencing Expression of NPR1 Did Not Result in TSWV Systemic Infection in Sw-5b-Transgenic N. benthamiana Plants

**Figure 4:**
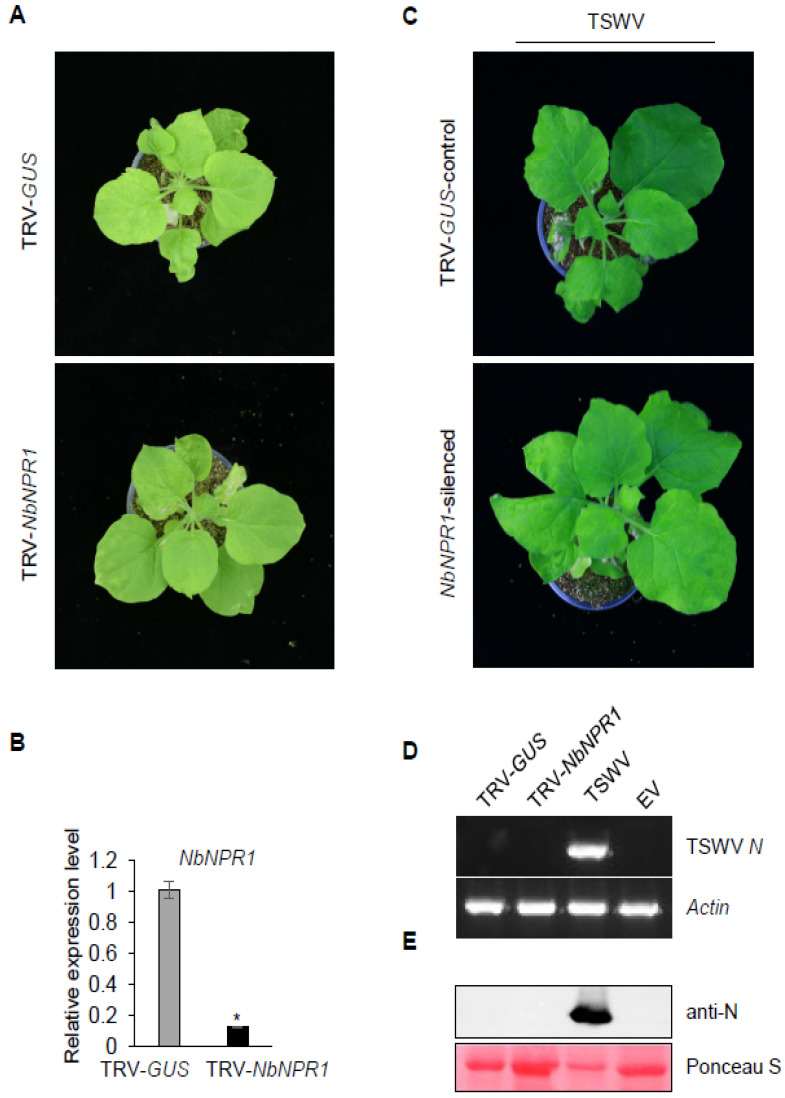
Analysis of the requirement for *NPR1* in *Sw-5b*-mediated resistance to TSWV. (**A**) TRV-*NbNPR1-* and TRV-*GUS*-treated *Sw-5b* transgenic *N. benthamiana* plants at 3 weeks post TRV agroinfiltration. (**B**) qRT-PCR analysis of the expression of *NbNPR1* mRNA in newly emerged leaves of *Sw-5b* transgenic *N. benthamiana* plant treated with *NPR1*-silenced transgenic plants. The *NbActin* and *NbEF1a* genes were used as internal controls. Student’s *t*-test was used for statistical analysis (* *p* < 0.05). (**C**) Analysis of systemic infection of TSWV in *Sw-5b*-transgenic *N. benthamiana* plants silenced for *NbNPR1.* TRV-*GUS* plants were used as a control. The gene-silenced newly emerged leaves of *Sw-5b*-transgenic *N. benthamiana* plants were inoculated with TSWV. The photographs of treated plants were taken at 14 days post TSWV inoculation. (**D**) RT–PCR detection of TSWV *N* RNA in systemic leaves of *Sw-5b*-transgenic *N. benthamiana* plants silenced for *NbNPR1* at 14 days post TSWV inoculation. A sample from TSWV-infected *N. benthamiana* tissues was used as a positive control. The *NbActin* gene was used as an internal reference gene. (**E**) Western blot analysis of TSWV N protein accumulation in the systemic leaves of *Sw-5b*-transgenic *N. benthamiana* plants silenced for *NbNPR1* in panel C using specific antibodies against TSWV N. A plant sample expressed with p2300 empty vector (Vec.) was used as a negative control. Ponceau S staining was used as a protein loading control.

NPR1 acts as a key regulator of SA-signaling pathways [33]. To examine whether NPR1 plays a role in *Sw-5b*-mediated resistance to TSWV, the mixed *Agrobacteria* carrying TRV1 and TRV-*NbNPR1* were co-infiltrated into *Sw-5b*-transgenic *N. benthamiana*. The *Agrobacterium* carrying the TRV-*GUS* was used as a control. At 3 weeks post TRV treatment, the growth state of transgenic *N. benthamiana* plants treated with TRV-*NbNPR1* was comparable to that of plants treated with TRV-*GUS* (Figure 4A and Figure S5). qRT-PCR assays confirmed that expression of *NbNPR1* mRNA was silenced in TRV-*NbNPR1*-treated plants (Figure 4B). The silenced plant leaves were inoculated with TSWV. At 14 days post TSWV inoculation, no obvious symptoms were observed in the systemic leaves of *NbNPR1*-silenced *N. benthamiana* plants (Figure 4C). RT-PCR and Western blot assays further confirmed the absence of TSWV in systemic leaves of *NbNPR1*-silenced *Sw-5b*-transgenic plants (Figure 4D,E). These data imply that silencing expression of *NbNPR1* did not result in TSWV systemic infection in *Sw-5b*-transgenic *N. benthamiana*.

### 3.5. The Helper NLRs NRC2/3/4 Were Required for Sw-5b-Mediated Systemic Resistance to TSWV Infection

**Figure 5:**
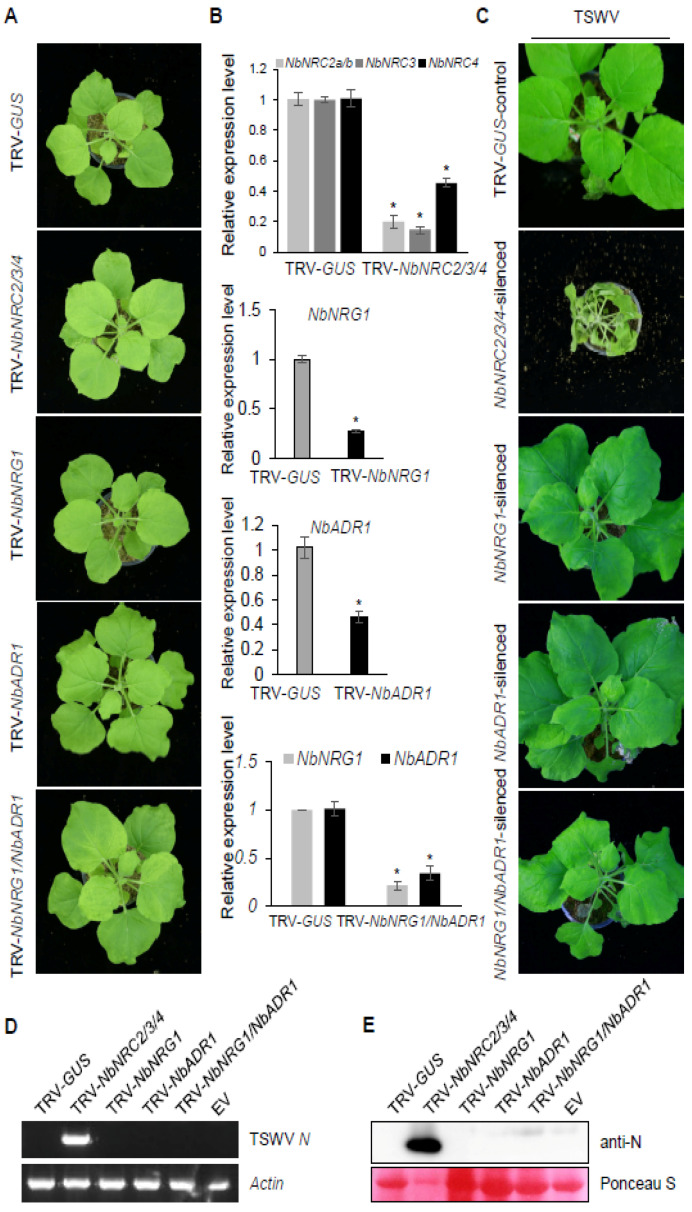
Requirement for the helper NLRs NRC2/3/4, NRG1, ADR1, and NRG1/ADR1 in *Sw-5b*-mediated resistance to TSWV infection. (**A**) TRV-based *NbNRC2/3/4*, *NbNRG1*, *NbADR1*, and *NbNRG1/NbADR1* gene-silenced plants at 3 weeks post TRV treatment. (**B**) qRT-PCR analysis of the expression of *NbNRC2a*, *2b*, *3*, and *4* or *NbNRG1* and *NbADR1* mRNA transgenic plants treated with TRV-*NbNRC2/3/4*, TRV-*NbNRG1*, TRV-*NbADR1*, and TRV-*NbNRG1/NbADR1*. The *NbActin* and *NbEF1a* genes were used as reference genes. Student’s *t*-test was used for statistical analysis (* *p* < 0.05). (**C**) Analysis of systemic TSWV infection of *Sw-5b*-transgenic *N. benthamiana* plants silenced for *NbNRC2/3/4*, *NbNRG1*, *NbADR1*, or *NbNRG1/NbADR1.* TRV-*GUS*-treated plants were used as a control. The gene-silenced newly emerged leaves of *Sw-5b*-transgenic *N. benthamiana* plants were inoculated with TSWV. The photographs of TSWV-challenged plants were taken at 14 days post TSWV inoculation. The systemic infection was evident in all 18 *NbNRC2/3/4*-silenced plants in three repeated experiments. (**D**) RT–PCR detection of TSWV *N* RNA in systemic leaves of plants in panel C at 14 days post TSWV infection. A sample from a TRV-*GUS*-treated plant was used as a negative control. The *NbActin* gene was used as an internal reference gene. (**E**) Western blot analysis of the accumulation of TSWV N protein in systemic leaves of plants in panel C using N-specific antibodies. The samples were harvested at 14 dpi. A plant leaf sample expressed with p2300 empty vector (Vec.) was used as a negative control. Ponceau S staining was used as a protein loading control.

In *N. benthamiana*, the NRC family contains NRC2a, NRC2b, NRC3, and NRC4. NRC2, NRC3, and NRC4 are functionally redundant. Simultaneous silencing of *NRC2*, *NRC3*, and *NRC4* expression in *N. benthamiana* suppressed the HR function of *Sw-5b* [29]. To examine the role of the helper NLRs NRC2/3/4 in systemic resistance mediated by *Sw-5b* to TSWV, we silenced *NbNRC2a*, *NbNRC2b*, *NbNRC3*, and *NbNRC4* simultaneously (*NbNRC2/3/4*) in *Sw-5b*-transgenic *N. benthamiana* by TRV-based VIGS (Figure 5A and Figure S6). The TRV-*GUS* vector was used as a control. At 3 weeks after TRV treatment, expression of *NbNRC2a*, *NbNRC2b*, *NbNRC3*, and *NbNRC4* was significantly reduced in the newly emerged leaves of *N. benthamiana* pretreated with TRV-*NbNRC2/3/4* (Figure 5B). These silenced leaves were challenged with TSWV. At 14 days post TSWV inoculation, TSWV was found to have infected the *NbNRC2/3/4*-silenced *Sw-5b*-transgenic *N. benthamiana* plants, causing systemic wilting (Figure 5C–E). These data imply that the *NbNRC2/3/4* genes are required for *Sw-5b*-mediated systemic resistance to TSWV infection, further supporting the essential role of helper NLR NRCs in downstream signaling of the sensor NLR *Sw-5b*.

### 3.6. Silencing Expression of the Helper NLRs NRG1 and ADR1 Did Not Affect Sw-5b-Mediated Systemic Resistance to TSWV

ADR1 and NRG1 play important roles in the regulation of ETI mediated by multiple NLRs [47,62]. To examine whether NRG1 and ADR1 are involved in *Sw-5b*-mediated resistance, we silenced *NbNRG1*, *NbADR1*, and both genes (*NbNRG1*/*NbADR1*) in *Sw-5b*-transgenic *N. benthamiana* plants using TRV-based VIGS. At 3 weeks post TRV treatment, the growth states of *NbNRG1*-, *NbADR1*-, and *NbNRG1*/*NbADR1*-silenced *N. benthamiana* plants were similar to that of TRV-*GUS*-treated plants (Figure 5A and Figure S6). The TSWV crude extract was inoculated onto the gene-silenced newly emerged plant leaves. At 14 days post TSWV inoculation, no systemic viral infection was found in systemic leaves of *NbNRG1*-, *NbADR1*-, or *NbNRG1*/*NbADR1*-silenced plants (Figure 5C). RT-PCR and Western blot assays confirmed the absence of TSWV in the systemic leaves of the *NbNRG1*-, *NbADR1*-, and *NbNRG1*/*NbADR1*-silenced plants (Figure 5D,E). These results imply that the helper NLRs NRG1 and ADR1 may not be involved in *Sw-5b*-mediated resistance.

## 4. Discussion

Here, we used the TRV-based VIGS system to characterize the roles of the SGT1/RAR1/HSP90, EDS1/NDR1, NPR1, and NRC/ADR1/NRG1 genes in *Sw-5b*-mediated resistance to TSWV. We found that chaperone SGT1 was essential for *Sw-5b* function; however, co-chaperone RAR1 was not. Suppression of both EDS1 and NDR1 did not affect *Sw-5b*-mediated resistance to TSWV. Moreover, suppression of NPR1 did not result in TSWV systemic infection in *Sw-5b*-transgenic plants. However, silencing the helper NLRs NRC2/3/4 compromised systemic resistance to TSWV infection in *Sw-5b*-transgenic *N. benthamiana* plants. Silencing another two helper NLRs, ADR1 and NRG1, did not affect *Sw-5b*-mediated immune signaling.

We explored the role of the SGT1–RAR1–HSP90 chaperone complex in *Sw-5b*-mediated immunity. Scofield et al. found that all three chaperone molecules, SGT1–RAR1–HSP90, were required for *Lr21*–mediated resistance. Silencing any of the three compromised resistance [63]. In our study, silencing *HSP90* resulted in significant morphological defects in the transgenic *N. benthamiana*, which led us to discontinue further investigation into the role of HSP90. Silencing *SGT1* compromised *Sw-5b* function; however, silencing *RAR1* did not. SGT1–RAR1–HSP90 forms a chaperone complex. However, *Sw-5b*-mediated immune signaling is independent of RAR1. A previous study on *Mi-1.2* also reported that *Mi-1.2*-mediated pest resistance required SGT1 but not RAR1 [64]. SGT1 has diverse roles in cellular pathways. For example, it plays an important role in nucleoplasm partitioning of tobacco *N* NLR [28]. We hypothesized that SGT1 may help in stabilizing *Sw-5b* immune receptor. In addition to functioning as a chaperone, SGT1 may play other roles in *Sw-5b*- and *Mi-1.2*-mediated defense signaling, and these roles of SGT1 may be independent of RAR1.

We tested the role of EDS1/NDR1 in *Sw-5b*-mediated resistance to TSWV. Early studies revealed that EDS1 was necessary for some CNLs. We found that the *Sw-5b*-mediated immune response was independent of EDS1. *Arabidopsis* CNLs such as RPS2, RPM1, and RPS5 are dependent on NDR1 for immunity. Our *Sw-5b*-mediated immunity was not dependent on NDR1. *Arabidopsis* CNLs, including HRT, ZAR1, RPP7, and RPP8, have also been found to be NDR1-independent [65]. In addition, the CNLs RPP13 and ZAR1 were independent of both EDS1 and NDR1 [66,67]. These findings imply that *Sw-5b* may induce EDS1- and NDR1-independent immune signaling pathways that remain to be uncovered.

We also explored the role of NPR1 in *Sw-5b*-mediated resistance. SA plays important roles in plant innate immunity and system acquired resistance (SAR). NPR1 acts as a receptor of SA and is a core player in SA signaling pathways. In our study, silencing expression of *NPR1* did not disrupt *Sw-5b* resistance to TSWV. However, NPR1 has been shown to be required for the function of the tobacco *N* gene. Our results imply that *Sw-5b*-mediated resistance may not rely on the *NPR1*-dependent SA signaling pathway.

Lastly, we tested the function of “helper” NLR proteins in *Sw-5b*-mediated systemic resistance to TSWV infection. NRCs are required for HR mediated by various NLRs in Solanaceae and disease resistance mediated by *Rpi-blb2* and *Rx* [29]. Here, we found that silencing the helper NLRs *NRC2/3/4* compromised *Sw-5b*-mediated resistance to TSWV, further supporting the essential role of NRC in NLR-mediated resistance in Solanaceae. ADR1 proteins are involved in both TNL- and CNL-mediated immunity [45,46,47,48]. NRG1 is a key component involved in TNL-mediated immune signaling pathways. ADR1 and NRG1 function redundantly in mediating NLR immune signaling [62]. Silencing *ADR1* and *NRG1* simultaneously partially compromised resistance to PVX [46]. We found that silencing *NRG1*, *ADR1*, or both had no effect on *Sw-5b*-mediated resistance to TSWV.

Mixed infection by two different viruses may have synergistic or suppressive effects. Our presented data are based on the use of TRV for gene silencing, followed by TSWV infection. We cannot rule out the possibility that infection of *Sw-5b* transgenic plants by TRV constructs may have enhanced or reduced the resistance to TSWV infection. In the future, gene knockout mediated by CRISPR/Cas9 in *Sw-5b* transgenic plant is needed to confirm the results obtained in this study.

In summary, we systematically investigated the requirement for the SGT1/RAR1/HSP90, EDS1/NDR1, NPR1, and NRC/ADR1/NRG1 genes in *Sw-5b* mediated immune signaling. We demonstrated that SGT1 and NRCs are conserved essential components of *Sw-5b*-mediated resistance to TSWV. Our results showing the independence of EDS1/NDR1 and NPR1 also indicate that *Sw-5b* has some unique and undiscovered defense signaling pathway that remains to be elucidated. As *Sw-5b*-mediated resistance is dependent on NRCs, we proposed that *Sw-5b* may either directly recruit downstream helper NLRs or indirectly transfer the upstream pathogen perception signals to downstream helper NLRs to amply and induce robust defense signaling.

## Data Availability

The data set analyzed for the current study is available from the corresponding author upon reasonable request.

## Supplementary Materials

The following are available online at https://www.mdpi.com/article/10.3390/v13081447/s1. Figure S1. Silencing *NbPDS* and *Sw-5b* by TRV-based VIGS in *Sw-5b*-transgenic *N. benthamiana* plants. Figure S2. *Sw-5b*-transgenic *N. benthamiana* plants infected with TRV-*GUS*, TRV-*NbSGT1*, and TRV-*NbRAR1* at 3 weeks post TRV treatment. Figure S3. Silencing the expression of *NbHSP90* in *Sw-5b*-transgenic *N. benthamiana* plants. Figure S4. *Sw-5b*-transgenic *N. benthamiana* plants infected with TRV-GUS, TRV- *NbEDS1*, and TRV-*NbNDR1* at 3 weeks post TRV treatment. Figure S5. *Sw-5b*-transgenic *N. benthamiana* plants infected with TRV-*GUS* and TRV-*NbNPR1* at 3 weeks post TRV treatment. Figure S6. *Sw-5b*-transgenic *N. benthamiana* plants infected with TRV-*GUS*, TRV-*NbNRC2/3/4*, TRV-*NbNRG1*, TRV-*NbADR1*, and TRV-*NbNRG1*/*ADR1* at 3 weeks post TRV treatment. Table S1. List of primers used in this study.
