# Supplementary material for: Characterization of the Roles of SGT1/RAR1, EDS1/NDR1, NPR1, and NRC/ADR1/NRG1 in Sw-5b-Mediated Resistance to Tomato Spotted Wilt Virus
Source: Viruses. 2021 Jul 25;13(8):1447. doi: 10.3390/v13081447 (PMC8402918; doi:10.3390/v13081447)
Supplement: Supplementary file 1 [file viruses-13-01447-s001.zip › viruses-1262534-supplementary/viruses-1262534-Figures S1-6.pdf]

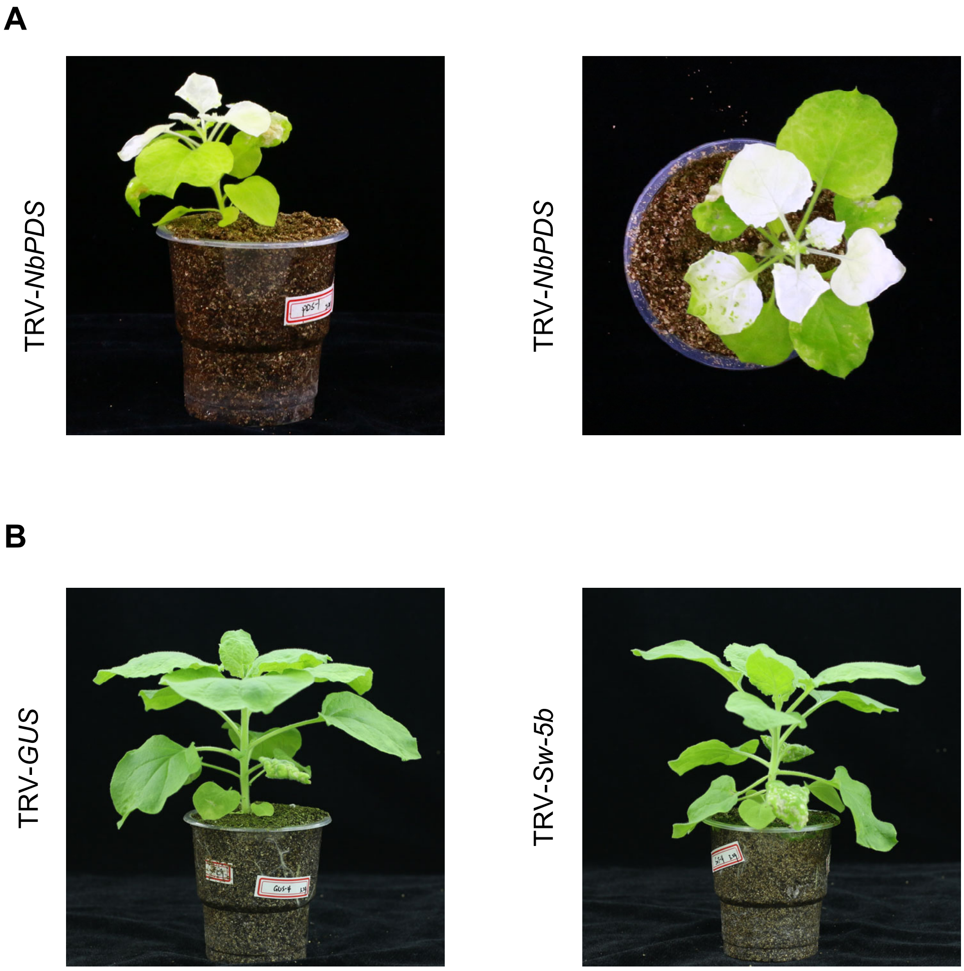

Supplemental Fig S1

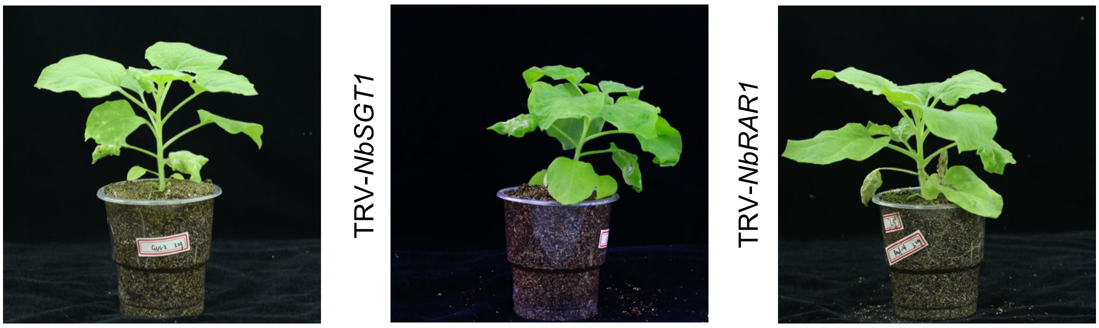

TRV-*GUS*

TRV-*NbSGT1*

TRV-*NbRAR1*

Supplemental Fig S2

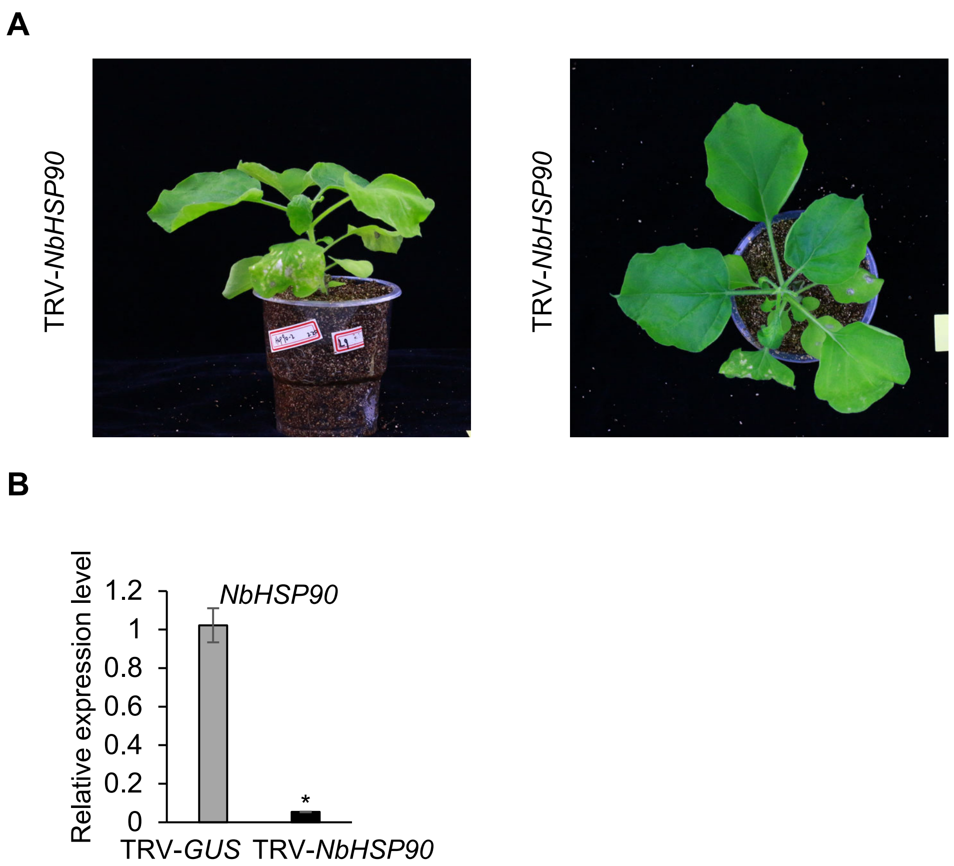

Supplemental Fig S3

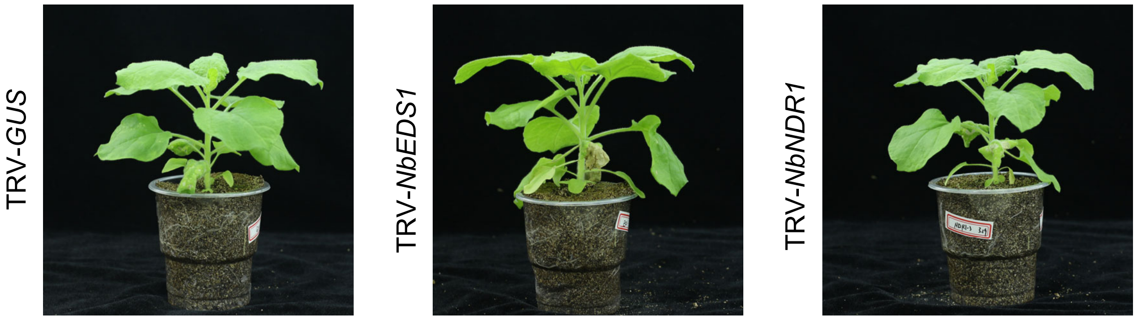

Supplemental Fig S4

TRV-*GUS*

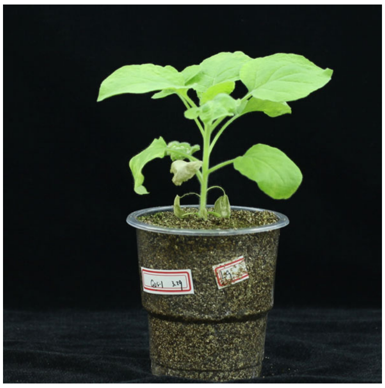

TRV-*NbNPR1*

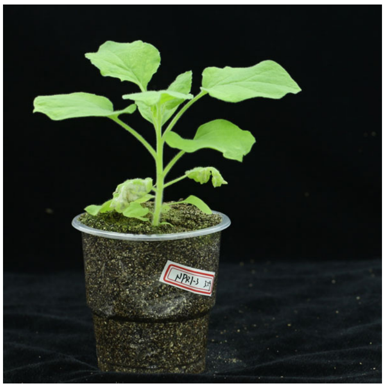

Supplemental Fig S5

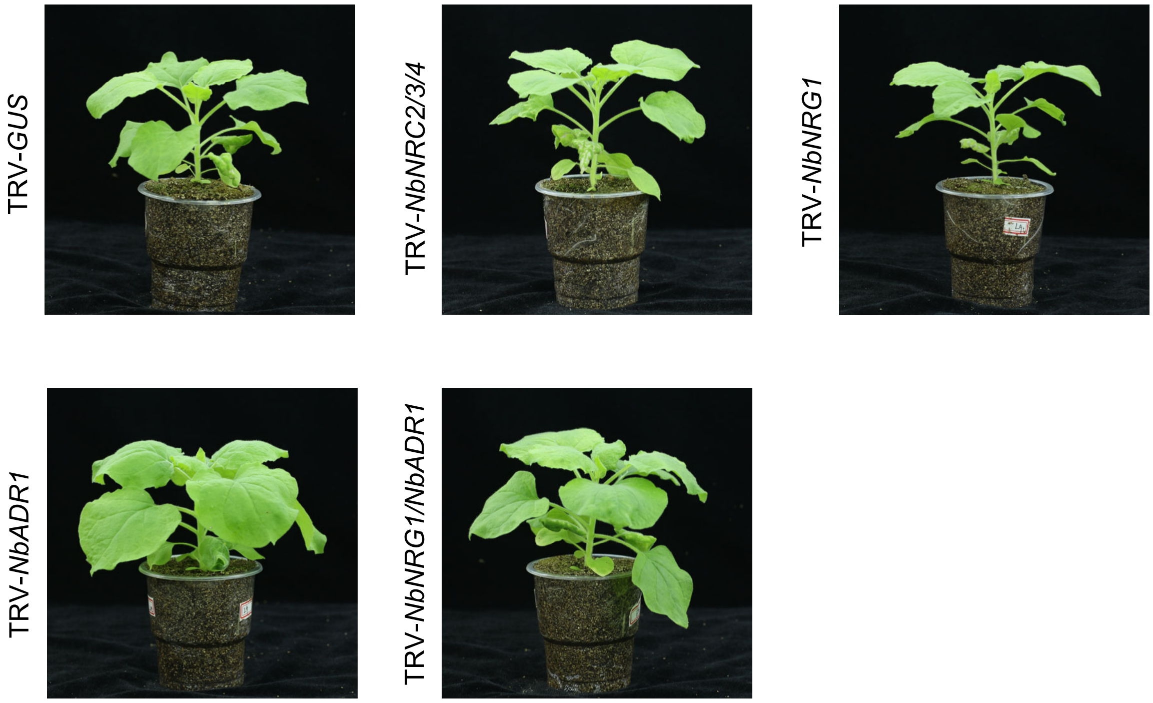

Supplemental Fig S6
