# Supplementary material for: Characterization of the Roles of SGT1/RAR1, EDS1/NDR1, NPR1, and NRC/ADR1/NRG1 in Sw-5b-Mediated Resistance to Tomato Spotted Wilt Virus
Source: Viruses. 2021 Jul 25;13(8):1447. doi: 10.3390/v13081447 (PMC8402918; doi:10.3390/v13081447)
Supplement: Supplementary file 1 [file viruses-13-01447-s001.zip › viruses-1262534-supplementary/viruses-1262534-Table S1.pdf]

Supplemental Table S1. List of primers used in this study.

Construct	Primers	Sequence (5'-3')	Purpose
pTRV2-*Sw-5b*	F	GTTCTAGATGGAAAATTTGAGGATATTAA	To amplify *Sw-5b* gene fragment, then clone into pTRV2.
	R	GTGGATCCTGGAATATCAGCAAAGCTAAG	
pTRV2-*NbRAR1*	F	GCTCTAGATGCCAGAGGATCGGTTGCAA	To amplify *NbRAR1* gene fragment, then clone into pTRV2.
	R	CGGGATCCGGTGATACGTTGGTCATAGAA	
pTRV2-*NbHSP90*	F	GCTCTAGAAATTTTTGTACCCCTACCAAGGTTA	To amplify *NbHSP90* gene fragment, then clone into pTRV2.
	R	CGGGATCCTGACAATCATTGACAGTGGTAT	
pTRV2-*NbEDS1*	F	CGTCTAGATGGATGCCCTCTTGTTGGT	To amplify *NbEDS1* gene fragment, then clone into pTRV2.
	R	CGGGATCCGTTCGTTTAAGCTTCTGGT	
pTRV2-*NbNDR1*	F	CGTCTAGAGCTAAGTCTAAGAACCACA	To amplify *NbNDR1* gene fragment, then clone into pTRV2.
	R	CGGGATCCTTCGGGGAAGTCCAAAAGT	
pTRV2-*NbNPR1*	F	GCTCTAGAGTAGTATGCCCCTCTCTTAG	To amplify *NbNPR1* gene fragment, then clone into pTRV2.
	R	CGGGATCCTGCCGGAGTTTGACTACTTC	
pTRV2-*NbNRC2/3/4*	F1	CCTCTAGAATGGCAGATGCAGTACTGAATTTTCCG	To amplify *NbNRC2a/b*, *NbNRC3* and *NbNRC4* gene fragments, then mix the three fragments and do overlap PCR using F1 and R3 to obtain *NbNRC2a/b*-*NbNRC3*-*NbNRC4* and then clone into pTRV2
	R1	CTACATCTGCTGCTACATCTGCCATGCAAGATTCCTGACTGTAGC	
	F2	GCTACAGTCAGGAATCTTGCATGGCAGATGTAGCAGCAGATGTAG	
	R2	GCGGATCCCTAGCAACATGAGTAAGATG	
	F3	CATCTTACTCATGTTGCTAGATGGCGAACGTTGCGGTGGAG	
	R3	GCGGATCCCTTAACATCCACAAATCTGCC	
pTRV2-*NbNRG1*	F	GCTCTAGATCGATGACATCGAGAG	To amplify *NbNRG1* gene fragment, then clone into pTRV2.
	R	CGGGATCCTCTTTCTCCTCAAGCA	
pTRV2-*NbADR1*	F	GCTCTAGATTAGGTGCTGGGATTGAGTT	To amplify *NbADR1* gene fragment, then clone into

	R	CGGGATCCATGATTGAAACCACGCGGAA	pTRV2.
pTRV2-*NbNRG1/NbADR1*	F1	GCTCTAGATCGATGACATCGAGAG	To amplify *NbNRG1* and *NbADR1* fragments, then mix the two fragments and do overlap PCR to obtain *NbNRG1-NbADR1* and clone into pTRV2
	R1	AACTCAATCCCAGCACCTAATCTTTCTCCTCAAGCA	
	F2	TGCTTGAGGAGAAAGATTAGGTGCTGGGATTGAGTT	
	R2	CGGGATCCATGATTGAAACCACGCGGAA	
Nbactin-1	F	ATGGCAGAAGGTGAGGAAATTCAGC	To detect the RNA level of *Nbactin* for semi-quantitative RT–PCR
	R	TTAGAAGCATTTTCTGTGAACAATT	
TSWV-*N*	F	ATGTCTAAGGTTAAGCTCAC	To detect the RNA level of TSWV-*N*.
	N	AGCAAGTTCTGCAAGTTTTG	
NbRAR1	F	GATGAGTTCATGAGCATATCGC	To quantify the RNA expression of *NbRAR1*.
	R	TCGTACCAATGTCCATACTAGC	
NbHSP90	F	TTTCGAATCCTCTCTGTGTTCA	To quantify the RNA expression of *NbHSP90*.
	R	GGAATGCAAACGTCTCTGTATC	
NbEDS1	F	TGGAATACTACATGCTCACGTT	To quantify the RNA expression of *NbEDS1*
	R	GTAATCCAAGTTTTGTCAGCGT	
NbNDR1	F	CAATTCATATTAACAGCAGG	To quantify the RNA expression of *NbNDR1*.
	R	GGAGTTGGAGTTATCAGAGT	
NbNPR1	F	ACATCAGCGGAAGCAGTAG	To quantify the RNA expression of *NbNPR1*.
	R	GTCGGCGAAGTAGTCAAAC	
NbNRC4	F	TGTGGTTCAGCATCTTTTTACG	To quantify the RNA expression of *NbNRC4*.
	R	CAAGCGAATCACTTTCCAAGAA	
NbNRC3	F	GGAAGGCGTGCTTCTTATATTG	To quantify the RNA expression of *NbNRC3*.
	R	ACAAGATCATTCAAGTGGTCCT	
NbNRC2a/b	F	AACAAGTTGGTGAAGTCAACAC	To quantify the RNA expression of *NbNRC2a/b*.
	R	AGTTCTGCACCAAAAATTCCAC	
NbNRG1	F	GCAAGCTTGGATCAGATTGTAG	To quantify the RNA expression of *NbNRG1*.
	R	AAAAGTAATCGGTATGCGAACG	
NbADR1	F	GGAGAAAATTGATATGCGGGAG	To quantify the RNA expression of *NbADR1*.
	R	TCTCAACATCATTCCACTGACA	
Nbactin-2	F	TTCTGTTCCAACCATCAATG	To quantify the RNA expression of *Nbactin* for qRT-PCR.
	R	TACCACCACTGAGGACAA	
NbEF1a	F	TGCTGCAACAAGATGGATGC	To quantify the RNA expression of *NbEF1a* for qRT-PCR.
	R	CCAGAGATGGGGACAAAGG	
